# Nicotinic-nAChR signaling mediates drug resistance in lung cancer

**DOI:** 10.7150/jca.36359

**Published:** 2020-01-01

**Authors:** Wan-Li Cheng, Kuan-Yuan Chen, Kang-Yun Lee, Po-Hao Feng, Sheng-Ming Wu

**Affiliations:** 1 Graduate Institute of Clinical Medicine, College of Medicine, Taipei Medical University, Taipei 11031, Taiwan; 2 Division of Pulmonary Medicine, Department of Internal Medicine, Shuang Ho Hospital, Taipei Medical University, New Taipei City 23561, Taiwan; 3 Division of Pulmonary Medicine, Department of Internal Medicine, School of Medicine, College of Medicine, Taipei Medical University, Taipei 11031, Taiwan

**Keywords:** nicotinic acetylcholine receptor, drug resistance, mitochondria, sirtuin, lung cancer

## Abstract

Lung cancer is the leading cause of cancer death worldwide. Cigarette smoking is the most common risk factor for lung carcinoma; other risks include genetic factors and exposure to radon gas, asbestos, secondhand smoke, and air pollution. Nicotine, the primary addictive constituent of cigarettes, contributes to cancer progression through activation of nicotinic acetylcholine receptors (nAChRs), which are membrane ligand-gated ion channels. Activation of nicotine/nAChR signaling is associated with lung cancer risk and drug resistance. We focused on nAChR pathways activated by nicotine and its downstream signaling involved in regulating apoptotic factors of mitochondria and drug resistance in lung cancer. Increasing evidence suggests that several sirtuins play a critical role in multiple aspects of cancer drug resistance. Thus, understanding the consequences of crosstalk between nicotine/nAChRs and sirtuin signaling pathways in the regulation of drug resistance could be a critical implication for cancer therapy.

## Introduction

Globally, lung cancer is greatest cause of cancer-related deaths. Lung cancer accounts for 14% and 12% of all cancers in men and women, respectively, and represents 24.6% of all cancer-related deaths 1. Because of its extraordinary disease burden and international variability in trends of population growth, aging, and smoking behaviors, the global epidemiology of lung cancer requires continual monitoring 2. Two main subtypes of lung cancer are small-cell lung carcinoma (SCLC) and non-SCLC (NSCLC), respectively accounting for 15% and 85% of all lung cancers 3. NSCLC is further classified into three types: squamous cell carcinoma (SCC), adenocarcinoma, and large-cell carcinoma. SCC is the subtype of NSCLC strongly correlated with cigarette smoking 4. NSCLC is a complex heterogenous disease with interpatient, intratumor, and inter-/intrametastatic heterogeneity at the subtype level 5. Both epithelial growth factor receptor (EGFR) mutation and gene amplification status may be notable in determining chemoresistance in NSCLC 6. Several studies have revealed that never-smokers with lung cancer are more responsive to EGFR-tyrosine kinase inhibitor (EGFR-TKI) therapy than smokers 7, 8. Tumor progression and chemoresistance mediated by nicotine and its metabolites have been partly attributed to phosphoinositide 3-kinase (PI3K)/AKT, nuclear factor-κB (NF-κB), and mitochondrial signaling pathways 7, 9, 10. Therefore, resistance is an inevitable barrier limiting lung cancer therapy effectiveness and reducing enthusiasm, making it today's pervasive challenge for long-term disease control.

## The biochemical and physiological properties of Nicotine

**Figure 1:**
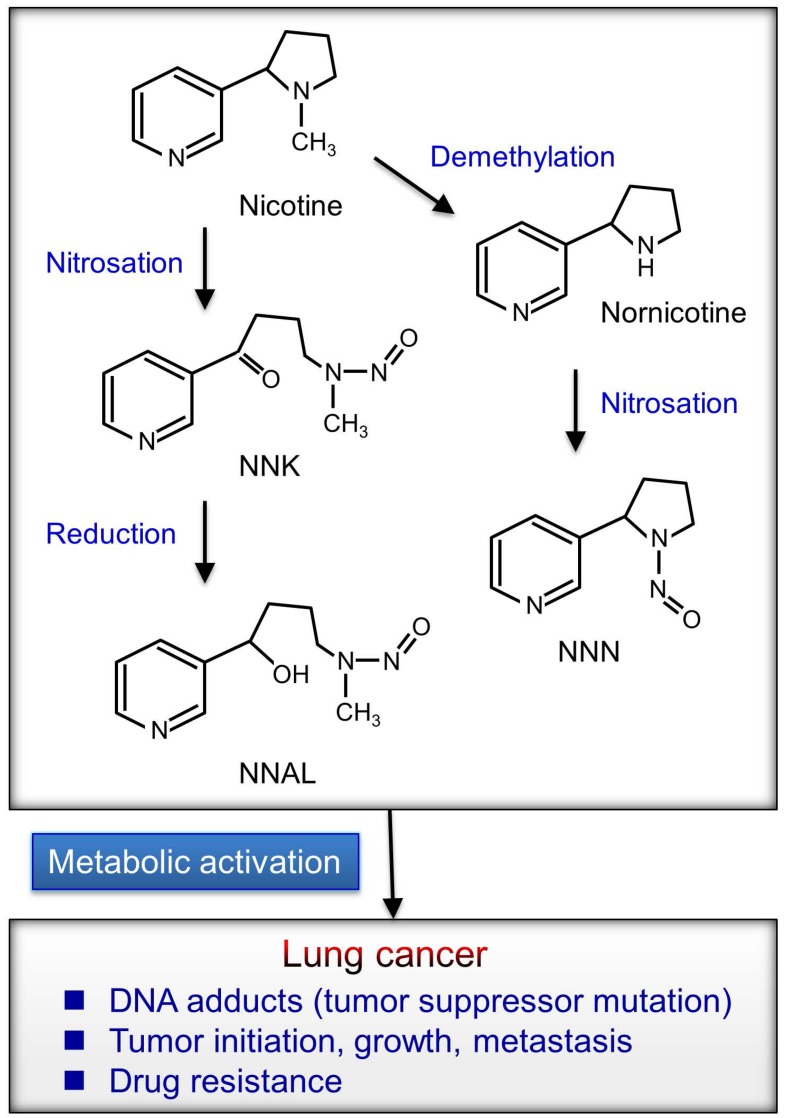
Tobacco-specific N-nitrosamines are formed by the N-nitrosation of nicotine. NNN (N′-nitrosonornicotine) and NNK (4-(metylnitrosamino)-1-(3-pyridyl)-1-butanon) are the most potential carcinogens formed by nicotine from cigarette smoke. NNAL (4-(methylnitrosamino)-1-(3-pyridyl)-1-butanol) is a metabolite from the reduction of NNK and total NNAL (NNAL and its glucuronides) in urine and can be used to examine the possible role of N-nitrosamines metabolites in tumor development. Nicotine metabolites carcinogens may induce multiple mutations in critical genes, such as p53, KRAS, p16, and Rb. A permanent mutation occurs in these critical genes that can contribute to activation of the oncogene or blockade of the tumor suppressor gene. Multiple aberrant events can continue to cause cells with abnormal regulation and eventually lung cancer progression.

Chemicals in cigarette smoke enter the bloodstream and affect the body; thus, smoking causes many diseases including cardiovascular disease, chronic obstructive pulmonary disease (COPD), and lung cancer 11. Furthermore, nicotine mediates therapeutic resistance, survival and antiapoptosis, and self-renewal of cancer stem cells and modulates many immune properties of cancer 12. Cigarette smoke contains several carcinogens including benzo[*a*]pyrene (BaP), polycyclic aromatic hydrocarbons (PAH), nicotine, and nitrosamines 4, 13-15. Nicotine can promote cancer progression by activating cell-surface receptors, particularly nicotinic acetylcholine receptors (nAChRs) and β-adrenergic receptors (β-AR) 16-18. Tobacco-specific N-nitrosamines is formed by the N-nitrosation of tobacco alkaloids (Fig. 1).

In addition to nicotine, its oncogenic derivatives 4-(methylnitrosamino)-1-(3-pyridyl)-1-butanone (NNK) and N-nitrosonornicotine (NNN), present in tobacco smoke, can activate nAChRs signaling and stimulate multiple cancer-promoting signaling 18, 19. Total 4-(methylnitrosamino)-1-(3-pyridyl)-1-butanol (NNAL), an NNK metabolite, is associated with the lung cancer risk in smokers 20. These oncogenic metabolites may induce the formation of DNA adducts that leads to mutations of tumor suppressor genes including Rb and p53 21. Nicotine or NNK signaling may contribute to cancer progression 18, 22. Nicotine was implicated in promoting the self-renewal of stem-like side-population cells from lung cancers. The subpopulation of cancer stem-like cells was implicated in tumor initiation, generation of heterogeneous tumor populations, metastasis, dormancy, and drug resistance 23. Furthermore, nicotine can inhibit apoptosis induced by opioids, etoposide, cisplatin, and UV irradiation in lung cancer cells 24-26. Therefore, the activation of nicotine signaling might be associated with drug resistance in lung cancer.

## The potential mechanisms of Nicotine on lung carcinogenesis

Cigarette smoke is associated with an increased risk of all histological types of lung cancer 27. In general, the lungs retain on average 60%-80% of mainstream smoke particulate matter and 90%-100% of nicotine after cigarette smoke inhalation 28. The nicotine-derived metabolites including NNK and NNN are potent carcinogens because they bind to α7nAChR 14. These metabolites and DNA interactions may be the primary cause of lung cancer in smokers. The binding activity of NNK to α7nAChR was 1,300 times greater than that of nicotine 29. Different nAChR subunits were expressed in NSCLC cells of smokers and nonsmokers 30. Nicotine present in the plasma of average smokers enhanced α7nAChR expression in human SCC cells 31. SCC was most strongly associated with cigarette smoke, and adenocarcinoma, the most common lung cancer in never-smokers, has the weakest association. The higher expression of all affected nAChR subunits (α7, dupα7, α5, and α9) in smokers than in nonsmokers indicated that tobacco components upregulate the expression of a few nAChR subunit genes in SCC histologic type 32. The literature has also indicated that only two subunits (α7 and α5) demonstrated significantly increased expression in SCC with a poor prognosis 32. Furthermore, patients with SCC who died presented significantly higher α7nAChR expression than patients with SCC who survived. For early-stage lung cancers, smoking cessation was associated with a large reduction in mortality risk 33. α5-nAChR was associated with lung cancer risk and onset, with exposure to a primary cancer etiologic factor (smoking duration and amount), and with the effects of a preventive action (smoking cessation) 34-37. Chronic exposure to nicotine and derivatives can lead to the upregulation of all nAChRs in smokers. Activation of nicotine-nAChR signaling promotes cancer progression 18. Upregulation of α7nAChR in lung cancer cells involved in the nicotine-induced tumor progression 38. Homomeric α7nAChR was implicated as the primary receptor facilitating nicotine- and NNK-mediated cell proliferation 21. The nicotine-α7nAChR axis can initiate cell invasion and the epithelial-to-mesenchymal transition (EMT) in NSCLC 39. nAChRs, β-AR, and EGFR often coexpress on human lung cancer cells and airway epithelial or endothelial cells that might lead to cancer progression 40. Nicotine signaling triggers the production of β-AR ligands, such as adrenaline and noradrenaline, which contribute to the development of lung cancer 21.

Survival analysis of a Cancer Genome Atlas (TCGA) lung cancer dataset demonstrated that high expression of acetylcholine receptors (AChRs) gene family such as CHRM2, CHRM3, CHRNA1, CHRNA2, CHRNA6, CHRNB3, or CHRNE is associated with favorable prognosis in NSCLC adenocarcinoma, but that of CHRNA5/α5nAChR or CHRNA7/α7nAChR is associated with an unfavorable prognosis 41. Among these proteins, only α7nAChR in the AChR family could affect prognosis in both lung adenocarcinoma and SCC 41. High CHRNA1 expression is associated with reduced survival in early-stage lung adenocarcinoma after complete resection 42. Notably, the levels of α7nAChR expression in SCC are higher than those in adenocarcinoma among patients with lung cancer, particularly in smokers 32. Thus, α7nAChR is a potential target for lung cancer treatment.

**Figure 2:**
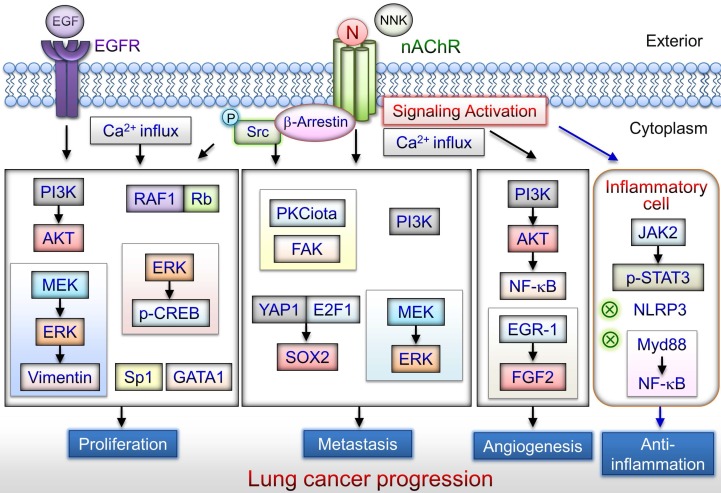
nAChR-mediated signaling pathways that lead to lung cancer progression. Nicotine/α7nAChR mediates the proliferative effects through several pathways including PI3K/AKT, MEK/ERK, RAF1/Rb, and Sp1/GATA1 activation signaling in lung cancer cells. Cigarette smoking is associated with metastasis of lung cancer. Nicotine/α7nAChR can induce NSCLC cell migration and invasion via the MEK/ERK signaling pathway. NNK enhances lung cancer cell migration via activation of ERK or the Src-PKCiota-FAK signaling axis. Nicotine/α7nAChR can enhance the metastasis of lung cancer cells through activation of the YAP1-E2F1 signaling axis. Moreover, α7nAChR may facilitate lung cancer progression including angiogenesis via the PI3K/AKT or ERG1/FGF2 signaling pathways. α7nAChR-mediated signaling may be a potential target for attenuating the production of inflammatory cytokines in inflammatory cells. Nicotine/nAChR can promote lung cancer development via different signaling pathways. N: nicotine; NNK: 4-(methylnitrosamino)-1-(3-pyridyl)-1-butanone.

Single nucleotide polymorphisms (SNPs) located on chromosome 15q25, which contains the nAChR subunits encoding by the CHRNA5, CHRNA3, and CHRNB4, are associated with lung cancer risk 43. Lung cancer risk was more than fivefold higher among individuals with both a family history of lung cancer and two copies of high-risk alleles rs8034191 or rs1051730 located in the 15q24-25.1 locus 44. The change in rs6495309T>C on 15q25 may affect the activity of OCT1 binding to the CHRNA3 promoter, which contributes to CHRNA3 overexpression. These results confirm that 15q25 is a susceptibility region for lung cancer in Chinese individuals 45. The gene cluster CHRNA5-CHRNA3-CHRNB4 contributes to nicotine dependence risk in African-American and European-American individuals 46. The missense SNP rs16969968 of CHRNA5 is significantly associated with both nicotine dependence and increased risk of lung cancer 47, 48. The genome-wide associations of CHRNA3 and CHRNA5 on 15q25.1 were confirmed in familial SCC 49. The CHRNA3 polymorphism functions as a genetic modifier of lung adenocarcinoma risk in the Chinese population, particularly in nonsmoking women 50. CHRNA5 polymorphism is associated with lung adenocarcinoma risk in a population-based series of lung adenocarcinoma patients and healthy controls. An analysis of a family-based series of nonsmoker lung cancer cases and healthy controls indicated a similar trend. In addition, the same D398N variation correlated with CHRNA5 mRNA levels in the lungs of patients with adenocarcinoma 51. Acetylcholine/nAChRs and muscarinic acetylcholine receptors can promote cancer cell and lung fibroblasts or myofibroblast proliferation, respectively 52, 53. Chronic nicotine inhalation or endogenous acetylcholine released in the lungs may play a role in lung disease including COPD and lung cancer. Nicotine/nAChRs stimulates several signaling pathways conferring lung cancer cell survival, as described in the next section (Fig. 2).

## The potential mechanisms of Nicotine on lung cancer progression

Nicotine may induce α7nAChR expression in human SCLC cells via the Sp1/GATA regulation signaling pathway 31. α7nAChR expression levels are elevated in SCC compared with adenocarcinoma of the lung, particularly in smokers 32, 54. High α7nAChR expression levels in lung cancer cells may be involved in the nicotine-induced tumorigenesis 32, 54. α7nAChR levels in patients with SCC who are active smokers are correlated with their smoking history 31. The function of α7nAChR-mediated lung cancer progression including in proliferation 31, 54-65, angiogenesis 66, and metastasis 39, 67-69, has been revealed (Fig. 2). The tumor-promoting effect is mediated by α7nAChR signaling pathways described in subsequent subsections.

### Cell proliferation

α7nAChR mediates the proliferative effects of nicotine in lung cancer cells 70. Nicotine/α7nAChR signaling enhances NSCLC cell proliferation by scaffolding protein β-arrestin-mediated activation of the Src and Rb-RAF protooncogene serine/threonine-protein kinase (RAF-1) pathways 55. Moreover, continuous exposure to nicotine in SCC of the lungs results in α7nAChRs upregulation, which may enhance tumor growth 31. Nicotine stimulates tumor growth and ERK activation in a murine orthotopic model of lung cancer 54. A blockade of α7nAChRs suppresses nicotine-induced lung cancer cell growth and vimentin expression through the MEK/ERK signaling pathway 63. Consistent with these results, nicotine increased expression of nAChR and stimulated proliferation of SCC cell line 65. α7nAChR may mediate the proliferative activity of nicotine in poorly differentiated NSCLC 56. Nicotine-induced α7nAChR and α4nAChR expression in NSCLC cells, along with p-CREB and p-ERK1/2 activation accompanied by increased noradrenaline, leading to cell proliferation 57. Nicotine/α7-nAChR promoted proliferation in human SCLC cells via the Sp1/GATA regulation signaling pathway 31. NNK/α7nAChR may increase cell growth in SCLC cells via an influx of Ca^2+^ 60. Nicotine stimulates NSCLC cell proliferation and PPARβ/δ expression through activation of PI3K/mTOR signals that suppress AP-2α binding activity to PPARβ/δ promoter 61. α7nAChRs or α9nAChRs mediated nicotine-induced cell proliferation and activation of the AKT and ERK signaling pathways 64. The activated cell-membrane α7nAChRs formed complexes with EGFR, whereas activated mitochondrial α7nAChRs was physically associated with the intramitochondrial protein kinases PI3K and Src that increased expression of cyclin D1 and activation of ERK1/2 lead to lung cancer proliferation 71. α-Cobratoxin, a high-affinity α7-nAChR antagonist reduced tumor growth in nude mice orthotopically engrafted with NSCLC cells 59. An α7nAChR inhibitor (APS8) may suppress NSCLCs proliferative effects of nicotine 58. These studies revealed that nicotine/α7nAChR signals mediate proliferation in lung cancer.

### Metastasis

Cigarette smoke status and history are associated with lung cancer metastasis 72, 73. α7nAChRs may mediate cancer cell growth depending on NSCLC differentiation status 56. α7nAChR and heteromeric nAChRs can promote tumor invasion in NSCLC 56. Nicotine/α7-nAChR can induce NSCLC cell migration and invasion via the MEK/ERK signaling pathway 39. NNK promotes lung cancer cell migration and contactin-1 expression via the α7nAChR-mediated ERK signaling pathway 67. Moreover, NNK enhances lung cancer cell migration and invasion via activation of the c-Src-PKCiota-FAK signaling axis 68. β-Cryptoxanthin may repress lung cancer cell motility through the downregulation of α7nAChR/PI3K signaling 69. Nicotine/α7nAChR signaling enhance migration and the expression of SOX2 in NSCLC cell lines through the YAP-E2F1 signaling axis 23. These studies suggest that α7nAChR can enhance lung cancer cell metastasis through the activation of different signaling pathways.

### Angiogenesis

α7nAChR enhances angiogenesis via the PI3K/AKT pathway and NF-κB activation, which is partially dependent on vascular endothelial growth factor (VEGF) 74. Nicotine/α7nAChR signaling mediates proangiogenic effects through angiogenesis and EMT 75. MG624, an α7nAChR antagonist, reduces nicotine-induced early growth response gene 1 (Egr-1) binding activity to the fibroblast growth factor 2 (FGF2) promoter that inhibits angiogenic effects in SCLC 66. Thus, α7nAChR may facilitate lung cancer progression including angiogenesis; however, its detailed effect requires investigation.

### Anti-Inflammation

α7nAChR attenuates ventilator-induced lung injury and plays an anti-inflammatory role in several inflammatory diseases 76-78. Activation of inflammation‐related receptors, such as toll-like receptors, enhances NF-κB signaling pathways in both acute and chronic inflammation that can considerably increase cancer risk 79. Choline/α7nAChR signaling modulates TNF release via the inhibition of NF‐κB activation 80. Moreover, nicotine/α7nAChR mediates anti-inflammatory action on macrophages via recruitment and activation of JAK2, initiating the STAT3 and SOCS3 signaling cascade 81. Nicotine/α7nAChR suppresses TNF-α expression in human airway epithelial cells by inhibiting MyD88 and NF-κB activity 82. An anti-inflammation study revealed that α7nAChR signaling inhibits NLRP3 inflammasome activation by preventing mitochondrial DNA release 83. Accumulating evidence suggests that the vagus nerve may modulate lung infection and inflammation through the α7nAChR signaling pathway 84. Thus, α7nAChR may be a potential target for attenuating inflammatory cytokine production in lung diseases 85.

Exposure to nicotine adversely affects dendritic cells, a cell type that has an important role in anticancer immunosurveillance 86. Nicotine may suppress anticancer immunity by increasing or reducing number of regulatory T cells and T helper 17 cells, respectively 87. Moreover, nicotine inhibits the cytotoxic activity of natural killer (NK) cells, and the effect of nicotine on NK cells can be abolished by β2nAChR deficiency 88. However, to understand immune regulation and progression in lung cancer, the roles of nicotine-mediated pathways in distinct immune cells warrant investigation.

## The potential mechanisms of sirtuins on cancer progression

Sirtuins play diverse roles in controlling the cell cycle and proliferation in response to stress, thus promoting cell survival, apoptosis, or senescence 89. Sirtuin 1 (SIRT1) has been involved in decision making over cellular senescence or apoptosis in lung diseases including COPD and cancer 53, 90. Cellular senescence is caused by replicative and stress-related senescence with p53 and p16 activation, respectively, leading to p21 activation and cell-cycle arrest. SIRT1 can function as p53 deacetylase that blocks p53-dependent pathways, thereby regulating cell-cycle and inactivating apoptotic process 91. SIRT1 and p53 play vital roles in maintaining genomic stability and integrity, which favor cell survival and protect against tumorigenesis. Increased DNA damage and cellular senescence may contribute to accelerated lung aging and COPD pathogenesis 53. SIRT6 can significantly suppress TGF-β-induced senescence of human bronchial epithelial cells via p21 degradation 92. Moreover, SIRT6 may play a pivotal role in inhibiting fibrosis. SIRT1 and SIRT6 prevent telomere dysfunction during DNA replication 93. SIRT1 and SIRT6 loss may promote aging and arise from oxidative stress through PI3K-mTOR signaling activation 94.

Sirtuins are responsible for cellular metabolic reprogramming and drug resistance by inactivating cell death pathways and promoting uncontrolled proliferation 90. SIRT1 overexpression is associated with poor prognosis in patients with lung cancer 95. SIRT1-mediated survival of cancer cells contributes to chemoresistance in tumors. Furthermore, SIRT1 is considerably expressed in the brain metastastic tissues of patients with NSCLC 96. SIRT1 may promote breast cancer progression through modulating AKT activity 97. SIRT1 was significantly overexpressed in human prostate cancer cells, and SIRT1 inhibition contributes to suppress cancer cell growth 98. SIRT1 limits prostatic intraepithelial neoplasia in SIRT1-knockout mice. In A/J mice, β-cryptoxanthin, strongly associated with reduced lung cancer risk, restores nicotine-reduced lung SIRT1 levels to that normal and inhibits nicotine-promoted lung tumorigenesis and emphysema 99. In addition, SIRT1 levels decrease in some human cancers including glioma, bladder, and ovarian cancer 100. SIRT1 also acts as a tumor suppressor via the c-Myc-SIRT1 feedback loop that regulates cell growth and transformation 101. Thus, the tumor suppressor or promoter role of sirtuins in cancer progression may depend on their tissue- and cancer-specific expression and the examined conditions. Similarly, SIRT6 plays functions of both tumor promoter and suppressor in the development of different cancer, potentially depending on specific tissue 90.

**Figure 3:**
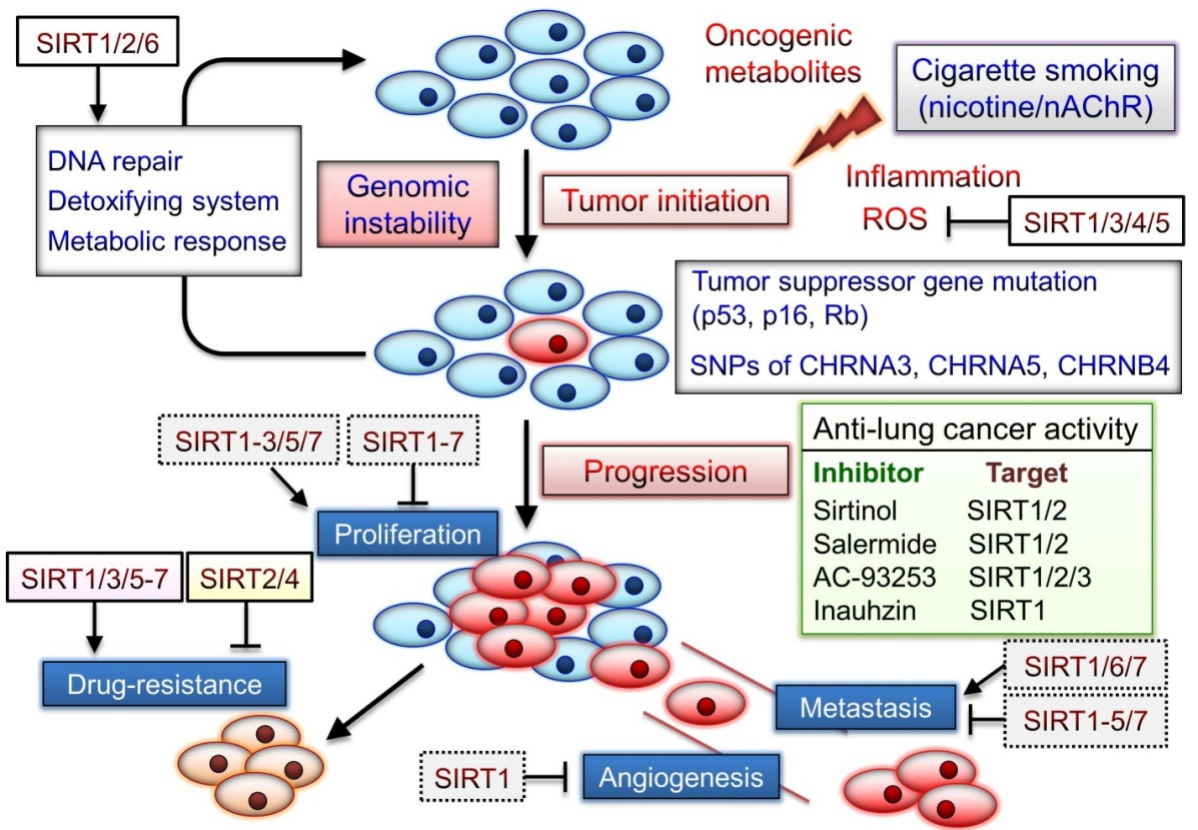
Roles of sirtuins may be involved in nicotine/nAChR-mediated signaling pathway. Cigarette smoke form carcinogens, including polycyclic aromatic hydrocarbons and the nicotine-derived nitrosamines 4-(methylnitrosamino)-1-(3-pyrydyl)-1-butanone (NNK) and N-nitrosonornicotine (NNN). Tumor suppressor mutations caused by these carcinogens may initiate carcinogenesis. NNK and NNN also significantly contribute to tumor development via activation of nAChRs signaling. SNPs located in a region of chromosome region 15q25 that contains nAChR subunits (CHRNA5, CHRNA3, and CHRNB4) are significantly associated with lung cancer risk. Sirtuins can exert their capacity to respond to environmental changes, and their expression is often altered in cancer. However, the tumor suppressor or promoter role of sirtuins in cancer progression may depend on their tissue- and cancer-specific expression and examined conditions. Several sirtuin inhibitors can suppress lung cancer development and blockade of sirtuins may be a potential anticancer strategy. The dotted box indicated the roles of sirtuins in other cancer types.

The SIRT1 inhibitor sirtinol induces senescence-like growth arrest through impaired activation of RAS-mitogen-activated protein kinase (MAPK) signaling in human lung cancer cells 102. The combination of inauhzin (SIRT1 inhibitor) and cisplatin or doxorubicin can considerably reduce NSCLC cell growth in a p53-dependent manner 103. Two SIRT2 inhibitors AEM1 and AEM2 can induce p53-dependent proapoptotic activity in NSCLC cells 104. Moreover, AEM2 demonstrates similar inhibition of SIRT2 as AC-93253. SIRT1/2 inhibitor, salermide, can increase death receptor 5 expression via the ATF4-ATF3-CHOP axis and contribute to NSCLC cell apoptosis 105. Therefore, sirtuins play notable roles in both lung cancer development and the nicotine/nAChR-regulated signaling (Fig. 3). Although several sirtuins can function as both tumor promoters and suppressors, SIRT1/3/5-7 blockade may aid in effective chemotherapy, as described in the next section.

## The potential mechanisms of Nicotine and sirtuins on drug resistance

Nicotine may reduce the cytotoxic effects of chemotherapy and radiotherapy that cause poor therapeutic response 87, 106. Nicotine-mediated tumor-promoting effects are apparently mediated by nAChRs expressed on cell membranes and by mitochondria 87. Additionally, mitochondria are critical mediators of cancer progression, as this process requires flexibility to adapt to cellular and environmental alterations in addition to cancer therapies 107. Nicotine-impaired metabolism and mitochondrial defects were critical in metabolic responses to cancer progression 108. Activation of cell-membrane and mitochondrial nAChRs produces a combination of growth-promoting and antiapoptotic signals that implemented the tumor-promoting action of nicotine in lung cells 71. Furthermore, nAChRs were identified to control either CaKMII or Src-dependent signaling pathways in mitochondria that protect cells from apoptosis 109.

Nicotine also permeated cells and activated mitochondrion-nAChRs coupling to inhibit mitochondrial permeability transition pore (mPTP) opening, preventing apoptosis 62. Nicotine-induced survival may occur by a mechanism of multisite phosphorylation of BAD, which may lead to human lung cancer and/or chemoresistance development 110. Activation of nicotine-α7nAChR signaling can trigger membrane depolarization, which activates voltage-gated calcium channels and subsequently activates the MAPK pathway, possibly increasing B-cell lymphoma-2 (Bcl-2) expression and apoptosis downregulation 111. Nicotine prevents cisplatin-mediated apoptosis by regulating α5nAChR/AKT signaling and several mitochondria proteins including Bcl-2, Bax, survivin, and caspase 3 in gastric cancer cells 112. Long-term nicotine exposure-induced chemoresistance is mediated by STAT3 activation and ERK1/2 downregulation via nAChR and β-AR in bladder cancer cells 113. Emerging evidence suggests that feedback activation of STAT3 signaling is a common cause of drug resistance to receptor tyrosine kinase-targeted therapies and conventional chemotherapy 114. Long-term exposure to NNK combined with arecoline activated EGFR/AKT signaling is involved in antiapoptosis, cancer stem cell properties, and cisplatin resistance in head and neck SCC (HNSCC) cells 115. Nicotine/α9nAChR-PPM1F signaling can attenuate p-p53 (Ser-20)- and p-BAX (Ser-184)-induced proapoptotic pathways 116. Therefore, nAChRs may be a promising molecular target to arrest lung cancer progression and reopen mitochondrial apoptotic pathways. Nicotine can induce erlotinib resistance via the crosstalk between α1nAChR and EGFR/AKT/ERK signaling pathways in NSCLC 117. A recent study suggested that smoking containing nicotine causes resistance to erlotinib therapy in NSCLC 118.

Sirtuins can exert their capacity to respond to environmental changes and their expression is often altered in cancer 89. SIRT1, SIRT3, SIRT4, and SIRT7 are strongly expressed in lung adenocarcinoma, whereas SIRT5 is highly expressed in SCC 119. Analysis of the TCGA NSCLC dataset revealed that high expression levels of SIRT2/6 were associated with longer overall survival (OS) 119. However, high SIRT6 expression was associated with poor OS in 98 patients with NSCLC 120. Nicotine enhanced oxidative stress and activates NF-κB 121. Several sirtuins are critical in inhibiting excessive, damaging levels of ROS that drive cancer drug resistance 122. Nuclear SIRT1 promotes ROS stress resistance via the deacetylation of several transcriptional regulators, including p53, forkhead homeobox type O (FOXO) proteins, PGC-1α, heat shock factor protein 1 (HSF1), and nuclear erythroid factor 2-related factor 2 (NRF2), and this contributes to antioxidant production 123. Studies have suggested that mitochondrial-sirtuins (SIRT3, SIRT4, and SIRT5) are members of a family of NAD^+^-dependent deacetylases and are implicated in the oxidative stress response through the regulation of mitochondrial metabolism and antioxidant mechanisms 124-126. Mitochondrial SIRT3 may coordinate ROS; SIRT5 also limits ROS by activating SOD1 and NRF2 to maintain cellular redox homeostasis 122, 126, 127. Thus, sirtuins may promote cancer cell survival by limiting ROS that would lead to cancer drug resistance. Several recent studies have supported the presence of sirtuins-mediated drug resistance with human cancers (Table 1). Some sirtuins regulate lung cancer progression, as described in the next subsection, and signaling molecules are associated with drug resistance.

### SIRT1

Nicotine can upregulate SIRT1 expression in a time- and concentration-dependent manner 128. BaP, a carcinogen in cigarette smoke, can induce SIRT1 in human bronchial epithelial cells 129. SIRT1 is involved in BaP-induced transformation associated with TNF-α-β-catenin axis activation and is a potential therapeutic target for lung cancer 129. The SIRT1-PARP-1 axis plays a critical role in the regulation of cigarette smoke-induced autophagy and has notable implications for understanding the mechanisms of cigarette smoke-induced cell death and senescence 130. α7nAChR-SIRT1 axis activation alleviates angiotensin II-induced VSMC senescence 131. Recent studies have focused on the biological functions of SIRT1 in metabolic diseases, cancer, aging and cellular senescence, inflammatory signaling in response to environmental stress, and cell survival 132-135. SIRT1 expression was a strong predictor for poor OS and progression-free survival in patients with NSCLC who underwent platinum-based chemotherapy 136. Silencing of SIRT1 could significantly enhance the chemosensitivity of lung cancer cells to cisplatin treatment 136. SIRT1 was negatively associated with proapoptotic factors BAD, BAX, and BID in TCGA NSCLC patients 119. SIRT1 suppression sensitizes lung cancer cells to WEE1 inhibitor-induced DNA damage and apoptosis 137. SIRT1 deacetylates and inactivates p53, allowing cells to bypass apoptosis 138. The transcription factor FOXO 3 alpha (FOXO3a) may induce the expression of several antioxidant genes, including Mn superoxide dismutase (MnSOD), catalase, peroxiredoxins 3 and 5 (Prx3 and Prx5, respectively), thioredoxin 2 (Trx2), thioredoxin reductase 2 (TR2), and uncoupling protein 2 (UCP-2) 139. SIRT1-mediated deacetylation of FOXO3a increases cell survival in response to oxidative stress 140. Moreover, SIRT1 may play a role in the acquisition of aggressiveness and chemoresistance in ovarian cancer and have potential as a therapeutic target for ovarian cancer 141.

### SIRT2-SIRT7

SIRT3, located in mitochondria, is correlated with NSCLC malignancy 142. α7nAChRs activation inhibits platelet-derived growth factor‐induced cells migration by activating the mitochondrial deacetylase SIRT3, implying a critical role for α7nAChRs in mitochondrial biology and PDGF‐related diseases 143. The activity of SIRT3 can protect cancer cells from chemotherapy-induced oxidative stress 144, 145. Additionally, SIRT3 promotes the activation of AKT signaling pathways in NSCLC 142. SIRT3 promoted p53 degradation in PTEN-deficient NSCLC cell lines via the ubiquitin-proteasome pathway 146. SIRT3 can also mediated FOXO3a nuclear translocation that activates MnSOD and catalase expression 147. Notably, SIRT1 knockdown cells can increase SIRT3 expression and cell survival and have relatively high resistance to H_2_O_2_ or etoposide treatment 148. SIRT3 might be a therapeutic target for breast cancer, improving the effectiveness of cisplatin and tamoxifen treatments 124. SIRT5 prevents cigarette smoke extract-induced apoptosis in lung epithelial cells via FOXO3 deacetylation 127. Besides, SIRT5 knockdown makes lung cancer cells more sensitive to drug (cisplatin, 5-fluorouracil or bleomycin) treatment 127. SIRT5 depletion suppresses the expression of NRF2 and its downstream drug-resistance genes 127.

Patients with high cytosol expression but low nuclear expression of SIRT6 can have poor clinical outcomes of lung cancer 120. Furthermore, SIRT6 knockdown in NSCLC cell lines can improve paclitaxel sensitivity by reducing NF-κB and Beclin1 (autophagy mediator) levels 120. Cigarette smoke increases SIRT6 expression *in vivo* (mice) and *in vitro* (rheumatoid arthritis synovial fibroblasts) 149. Reduced SIRT6 expression mediates the augmentation of radiation-induced apoptosis via cAMP signaling in lung cancer cells 150. A recent report revealed that SIRT7 depletion promotes gemcitabine-induced cell death 151. Functioning as an oncogene, SIRT7 can be suppressed by miR-3666, which could increase NSCLC cell apoptosis 152.

Thus, the aforementioned studies together have demonstrated tumor progression modulated by the SIRT1, SIRT3, and SIRT5-7, along with the tumor-suppressive effects of SIRT2 and SIRT4. SIRT2 mediates the ROS production and p27 levels, leading to lung cancer cell apoptosis and cell-cycle arrest 153. SIRT2 overexpression increases NSCLC cells' sensitivity to cisplatin treatment 153. Moreover, recent findings suggest that SIRT4 inhibits lung cancer progression through mitochondrial dynamics mediated by the ERK-Drp1 pathway 154. At present, one clinical trial (NCT02416739) is studying the combinatorial effects of the human sirtuin inhibitor (nicotinamide) and EGFR-TKI in NSCLC. The discovery of specific SIRT regulation and EGFR-TKI treatment would help elucidate the roles of sirtuins in lung cancer development. Although sirtuin clearly is critical in carcinogenesis, the crucial mechanisms by which the nicotine-mediated signaling or specific sirtuin pathways in different cell context lead to drug resistance require elucidation.

**Figure 4:**
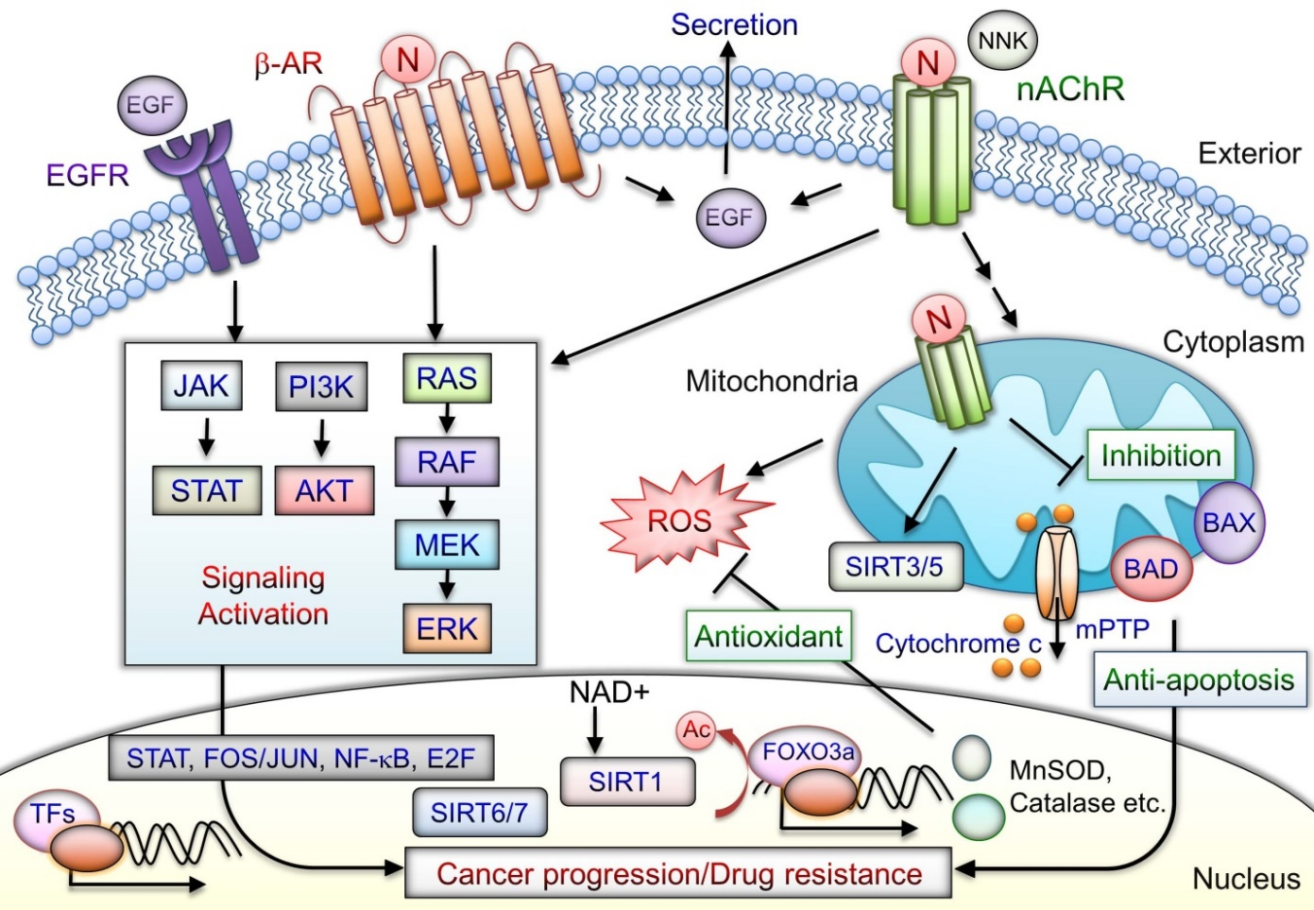
Schematic of mediation of tumor-promoting actions by nicotine/nAChR. Nicotine interacts with nAChR and stimulates activation and crosstalk with β-AR and EGFR downstream, signaling to promote cancer progression. Activation of nAChRs and β-AR mediates EGF secretion to further transactivate EGFRs. In cancer cells, the signaling pathways downstream of nAChRs promote drug resistance and antiapoptosis by activating the transcription factors including STAT, NF-κB, Jun/Fos, and E2F through JAK, PI3K/AKT, RAS, RAF, and the MAPK signaling cascade. Mitochondrial nAChRs trigger phosphatidyl-inositol-3-kinase (PI3K) and AKT signaling pathways that prevent mPTP opening and cytochrome c release. Nicotine-induced antiapoptosis and drug resistance may include several mechanisms involved in overexpression of sirtuin proteins, phosphorylation of BAD, and blockade of BAX translocation, leading to tumor cell development. SIRT3 and SIRT5 are mitochondrial proteins. SIRT6 and SIRT7 are localized in the nucleus. SIRT1-mediated deacetylation of FOXO3a can induce expression of antioxidant enzymes including MnSOD and catalase that increase cell survival during cellular oxidative stress. Consequently, nicotine/nAChR mediates antiapoptotic pathways and concurrently crosstalks with β-AR or EGFR signaling activation may lead to cancer progression. N: nicotine; Ac: acetylation; NNK: 4-(methylnitrosamino)-1-(3-pyridyl)-1-butanone.

Cell-membrane nAChRs implement upregulation of proliferative and survival genes 62. Nicotine can promote oral precancerous growth through suppression of apoptosis by upregulating α7nAChR and peroxiredoxin 155. α7nAChR-mediated cell protection, through JAK2/PI3K/AKT/signal transducer and activator of transcription 3(STAT3)/NF-κB activation, leads to Bcl-2 production 156. Nicotine binds to nAChRs and stimulates secretion several factors including epidermal growth factor (EGF), VEGF, and neurotransmitters 157. Nicotine/nAChRs mediates EGF secretion and subsequent EGFR signaling activation, thus contributing to antiapoptosis 18. Nicotine and NNK also bind to β-ARs and promote survival signaling cascades 18, 30. Moreover, tissue-specific expression of α7β2, α3β2, α3β4, and α4β2 nAChRs located in the mitochondria outer membrane with anion channels that regulate the release of proapoptotic cytochrome c or ROS production has been observed 78, 158, 159. nAChR signaling in mitochondria is stimulated and engages PI3K/AKT kinases, similar to those activated by plasma membrane nAChRs. Nicotine contributes to progression and erlotinib resistance in an NSCLC xenograft model through the nAChR-EGFR cooperation 117. The nicotine-mediated α5nAChR/AKT signaling pathway prevents cisplatin-induced cancer cell apoptosis 112. Blockade of α7nAChRs inhibited nicotine-induced tumor growth and vimentin expression in NSCLC through the RAS-RAF-MAPK kinase (MEK)-extracellular signal-regulated kinase (ERK) signaling pathway 63. The nicotine and derivatives may mediate oncogenic signaling via nAChR, β-AR, and EGFR and combined with the effects of antiapoptosis in mitochondria that contribute to cancer progression (Fig. 4). The nicotine/nAChR signaling crosstalk with SIRT1/3/5-7 may contribute to cancer drug resistance.

## Conclusions and Future Directions

Genome-wide association studies have indicated a strong link between nicotine/nAChRs and lung cancer risk 34, 38. Nicotine might lead to suppressed apoptosis and cisplatin resistance via α5nAChR/AKT signaling 112. In addition, α7nAChR may be implicated in the NAD^+^/SIRT1 pathway, which promotes chemotherapeutic drug resistance 131. Nicotine/α9nAChR signaling can reduce apoptotic pathways 116. Nicotine-mediated cancer progression is mediated by nicotine/nAChRs signaling. By contrast to the tumor-suppressing role of SIRT2/4, the roles of SIRT1/6/7 and mitochondrial SIRT3/5 are strongly associated with drug resistance in lung cancer 119, 120, 124, 135, 142, 144, 146, 150-154. However, SIRT1/2 inhibitor, sirtinol, can increase anticancer potential and chemosensitivity in several cancer cells 102, 160, 161. SIRT1 may have contradictory roles in promoting or suppressing in different cell contexts or types of tumor development 162. Moreover, shikonin causes apoptosis in some lung cancer cell lines via the FOXO3a/EGR1/SIRT1 signaling pathway activation 163. In contrast to the tumor-suppressing role of SIRT1, the role of shikonin involves promoting liver cancer cell death by downregulating the SIRT1-MDR1/P-gp signaling pathway 164. However, emerging evidence suggests that SIRT1 can promote cancer drug resistance (Table 1). Thus, an investigation to unravel cancer cellular contexts in which ROS are beneficial or harmful, with sirtuins having tumor-suppressing or promoting functions, would contribute to the literature. Thus, a series of more comprehensive studies are necessary to validate the underlying molecular mechanisms of the nicotine-mediated pathway with specific sirtuin and related targets in different types of cancer.

Nicotine activates α7nAChR and β2nAChR, and these two factors are associated with EGF and VEGEF receptors, respectively. However, activated mitochondrial α7nAChRs and β4nAChR interacted with the PI3K and Src 62. This interaction leads to increased cell proliferation and ROS-mediated apoptosis resistance through ERK signaling and mPTP inhibition, respectively 62. The same group of researchers demonstrated a nicotine-mediated growth-promoting effect via interaction of α7nAChR with that EGF, α3nAChR with VEGF, α4nAChR with insulin-like growth factor I (IGF-I) and VEGF, whereas α9nAChR was with EGF, IGF-I, and VEGF. Similar to the binding affinity of cell-membrane nAChRs, nicotine can bind to mitochondrial nAChRs. Mitochondrial nAChRs coupled with the inhibition of mPTP opening causes tumor progression 71. These results indicate that nicotine-mediated activation of the cell-membrane or mitochondrial nAChRs leads to tumor-promoting and antiapoptotic signals in tumor development. Therefore, nAChRs may be a potential molecular target to suppress lung cancer progression and trigger mitochondrial apoptotic pathways.

An accumulating body of evidence is demonstrating that long noncoding RNAs (lncRNAs) have various biological functions, including modulation of growth, cell differentiation, drug resistance, and cancer progression 165-167. MALAT-1 is highly expressed and mediates poor progression in lung cancer 168. LncRNA-SCAL1 (smoke and cancer-associated lncRNA1) can alleviate CS-mediated oxidative stress in airway epithelial cells 169. Although lncRNAs were to be dysregulated in various human diseases, how lncRNAs connect with environmental exposures remains largely unknown. GAS5 may be proapoptotic and increase sensitivity to cell death due to environmental stressors 170, 171. HOTAIR mediates EMT because of cigarette smoke extracts 172, and similar studies have indicated that MALAT1 is involved in cigarette smoke extract-induced EMT and malignant transformation 172. Circulating extracellular vesicles (EVs) can transfer biomolecules, including ligands, cytokines, and genetic information, to recipient cells to enact functional changes 173. EVs can contain relatively stable RNA species, including small noncoding RNAs and lncRNAs 174. LncARSR is highly expressed in sunitinib-resistant renal cancer cells, and these drug-resistant cancer cells can transfer lncARSR to drug-sensitive recipient cells and acquire chemoresistance via exosomes 175. Exosomes mediated transfer of lncRNA UCA1 can considerably increase tamoxifen resistance in estrogen receptor (ER)-positive breast cancer cells 176. Thus, lncRNAs may cause cancer-acquired chemoresistance via exosomal pathway. However, whether nicotine promotes cancer drug resistance via the tumor environment communication remains unclear. Thus far, nicotine-mediated drug resistance induced by lncRNAs in lung cancer remains unclear. Therefore, nicotine-mediated drug resistance through lncRNA regulation in NSCLC warrants further exploration.

Over the past few years, nAChRs have been found to be selectively overexpressed in various cancers, including lung cancer. Targeting nAChRs signaling pathways can significantly attenuate nicotine-associated drug resistance. Several α7nAChR antagonists are potential anticancer drugs 38. Collectively, studies have suggested that nAChR-induced antiapoptotic effects may play notable roles in drug resistance in lung cancer. Elucidation of the consequences of nicotine/nAChRs signaling crosstalk with other pathways and specific sirtuins may be crucial to therapeutic implications. These studies may facilitate the design of useful therapeutic strategies to reverse chemoresistance in patients with different types of cancer.

## Implication

Nicotine and derived metabolites are associated with lung cancer risk in smokers. The α7nAChR is significantly upregulated in NSCLC and correlates with its unfavorable prognosis. Activation of nicotine/α7nAChR signaling leads to lung cancer progression. Studies have suggested that nicotine/nAChR axis and SIRT1/3/5-7 mediates cancer drug resistance. Blockade of the signaling mediated by nicotine/nAChR and specific sirtuins may enhance the efficacy of chemotherapy.

## Figures and Tables

**Table 1:** Effects of sirtuins on cancer drug resistance

Types of sirtuin	Types of cancer	Drug	Effect of cancer drug resistance	Year of publication	Ref
SIRT1	CRC	TRAIL	miR-128 suppressed SIRT1 expression to sensitize TRAIL-induced apoptosis	2018	177
SIRT1	Breast cancer	Paclitaxel	A SIRT1-PRRX1-KLF4-ALDH1 circuitry regulates breast cancer stemness and metastasis. KLF4 inhibitor Kenpaullone sensitizes breast cancer cells	2018	178
SIRT1	Cervical cancer	Paclitaxel	Knockdown of SIRT1 promotes apoptosis of paclitaxel-resistant human cervical cancer cells	2018	179
SIRT1	ATC	Doxorubicin	6-phosphogluconate dehydrogenase (6PGD) was critically involved in ATC resistance to doxorubicin. Decreased enzymatic activity of SIRT1 in response to 6PGD inhibition in doxorubicin-resistant ATC cells	2018	180
SIRT1	Cervical cancer	Doxorubicin	β2-AR activation induces chemoresistance by modulating p53 acetylation through upregulating Sirt1 in cervical cancer cells	2017	181
SIRT1	Gastric cancer	Cisplatin	miR-132 regulated SIRT1/CREB/ABCG2 signaling pathway contributes to cisplatin resistance	2017	182
SIRT1	HCC	Oxaliplatin	LncRNA HULC triggered autophagy by stabilizing SIRT1 and attenuates chemosensitivity of HCC cells	2017	183
SIRT1/SIRT3	Breast and Cervical cancer	Etoposide	Cancer cells with low SIRT1 levels maintained their resistance and survival by increasing SIRT3 expression	2017	148
SIRT1	Prostate cancer	Docetaxel	The UCA1-miR-204-SIRT1 axis modulates docetaxel sensitivity of prostate cancer cells	2016	184
SIRT1	Breast cancer	Tamoxifen	Brachyury mediates tamoxifen resistance by regulating SIRT1	2016	185
SIRT1	Bladder cancer	Capsaicin	Capsaicin inhibited multiple bladder cancer cells by inhibiting tumor-associated NADH oxidase (tNOX) and SIRT1	2016	186
SIRT1	ATL	Etoposide	SIRT1 inhibition enhances chemosensitivity and survival of ATL cells by reducing DNA double-strand repair	2015	187
SIRT1	Endometrial carcinoma	Cisplatin and paclitaxel	SIRT1 overexpression significantly enhanced drug resistance. Selective SIRT1 inhibitor (EX527) significantly increased chemosensitivity	2015	188
SIRT1	CML	Hsp90 inhibitors (17-AAG and AUY922)	SIRT1 inhibitors (amurensin G and EX527) effectively potentiated sensitivity of Hsp90 inhibitors	2015	189
SIRT1	ESCC	Cisplatin	Overexpression of SIRT1 may cause resistance of ESCC cells to cisplatin through Noxa expression	2015	190
SIRT1	PC	5-fluorouracil (5-FU) and gemcitabine	Overexpression of miR-494 inhibited chemoresistance of PC by downregulating SIRT1 and c-Myc	2015	191
SIRT1	CRC	5-FU	SIRT1/PGC1α-dependent increase in oxidative phosphorylation leads to CRC drug resistance	2015	192
SIRT1	CML	Imatinib	Divalproex sodium enhances antileukemic effects of imatinib in CML through SIRT1	2015	193
SIRT1	AML	TKI (Quizartinib, AC220)	Inhibition of SIRT1 by SIRT1 inhibitor Tenovin-6 (TV6) enhanced TKI-mediated sensitivity	2014	194
SIRT1	Thyroid cancer	Etoposide	SIRT1-Foxp3 signaling confers drug resistance	2014	195
SIRT1	Breast cancer	TRAIL	Metformin mediates miR-34a to suppress the SIRT1/PGC-1α/NRF2 pathway and increases drug sensitivity	2014	196
SIRT1	CML	TKIs (imatinib, nilotinib or dasatinib)	All-trans-retinoic acid (ATRA) effectively blocked acquisition of BCR-ABL mutations and resistance. ATRA inhibited NAD+-dependent SIRT1 deacetylase via CD38 expression	2014	197
SIRT2	RCC	5-FU	SIRT2+ cells mediates RCC drug resistance	2018	198
SIRT2	AML	Daunorubicin, arabinocytidine	SIRT2 mediates multidrug resistance in AML cells via ERK1/2 signaling pathway	2016	199
SIRT2	Melanoma	Doxorubicin	AC-93253, a SIRT2 inhibitor increases drug sensitivity	2015	200
SIRT3	Synovial sarcoma	Pazopanib	Knockdown of SIRT3 confers increased resistance to chemotherapeutic agents	2018	201
SIRT3	HCC	Sorafenib	SIRT3 protein expression was significantly higher in patients treated with metformin	2017	202
SIRT3	Glioma	Linalool	Overexpression of SIRT3 significantly inhibited a linalool-induced increase of mitochondrial ROS production and apoptotic cell death	2017	203
SIRT3	Breast cancer	Cisplatin	SIRT3 silencing sensitizes breast cancer cells to cytotoxic treatments through ROS production	2017	124
SIRT4	CRC	5-FU	SIRT4 increased the sensitivity of CRC cells to 5-FU	2016	204
SIRT5	NSCLC	CDDP, 5-FU or bleomycin	SIRT5 facilitates cancer cell growth and drug resistance in NSCLC cells	2014	127
SIRT6	HCC	Doxorubicin	SIRT6 increased doxorubicin resistance via FOXO3 activity	2018	205
SIRT6	NSCLC	Gefitinib	Astragaloside IV sensitizes NSCLC cells to gefitinib potentially via regulation of SIRT6	2017	206
SIRT6	PC	Gemcitabine	Quinazolinedione SIRT6 inhibitors sensitize cancer treatment	2015	207
SIRT6	NSCLC	Paclitaxel	SIRT6 knockdown NSCLC cells improved drug sensitivity	2015	120
SIRT6	Breast cancer	Trastuzumab (Herceptin)	MDM2-mediated degradation of SIRT6 phosphorylated by AKT1 promotes drug resistance	2014	208
SIRT7	NSCLC	Gemcitabine	Depletion of SIRT7 promoted drug sensitivity	2018	151
SIRT7	Breast cancer, osteosarcoma, and ovarian cancer	Cisplatin, Doxorubicin	SIRT7 inhibition significantly increases stress resistance and modulates insulin/IGF-1 signaling pathways	2014	209

CRC, Colorectal cancer; ATC, Anaplastic thyroid carcinoma; HCC, Hepatocellular carcinoma; ATL, Adult T-cell leukemia-lymphoma; CML, Chronic myeloid leukemia; ESCC, Esophageal squamous cell carcinoma; PC, Pancreatic cancer; AML, Acute myeloid leukemia; RCC, Renal cell carcinoma; and NSCLC, Non-small-cell lung carcinoma.

## Abbreviations

SCLC: small-cell lung carcinoma
NSCLC: non-small-cell lung carcinoma
EGFR: epithelial growth factor receptor
EGFR-TKI: EGFR-tyrosine kinase inhibitor
NF-κB: nuclear factor-κB
PI3K: phosphoinositide 3-kinase
BaP: benzo[*a*]pyrene
PAH: polycyclic aromatic hydrocarbons
nAChRs: nicotinic acetylcholine receptors
β-AR: β-adrenergic receptors
NNK: 4-(methylnitrosamino)-1-(3-pyridyl)-1-butanone
NNN: N-nitrosonornicotine
NNAL: 4-(methylnitrosamino)-1-(3-pyridyl)-1-butanol
SNP: single nucleotide polymorphism
RAF-1: proto-oncogene serine/threonine-protein kinase
MEK: mitogen-activated protein kinase kinase
ERK: extracellular signal-regulated kinases
MAPK: mitogen-activated protein kinase
EMT: epithelial-to-mesenchymal transition
SOX2: sex-determining region Y-box 2
VEGF: vascular endothelial growth factor
Egr-1: growth response gene 1
FGF2: fibroblast growth factor 2
STAT3: signal transducer and activator of transcription 3
NLRP3: nucleotide-binding domain and leucine-rich repeat containing protein 3
ASC: adult stem cell
ROS: reactive oxygen species
NAC: N-acetyl-cysteine
mPTP: mitochondrial permeability transition pore
Bcl2: B-cell lymphoma-2
HNSCC: head and neck squamous cell carcinoma
SIRT1: Sirtuin 1
TAM: tumor-associated macrophage
FOXO3: Forkhead box 3
NRF2: nuclear factor (erythroid-derived 2)-like 2
OS: overall survival
RFS: recurrence-free survival
and NRF2: nuclear factor erythroid-2-related factor
